# High flow versus conventional nasal cannula for oxygenation and ventilation maintenance during surgery with intravenous deep sedation by propofol: a randomized controlled study

**DOI:** 10.1186/s12871-021-01432-4

**Published:** 2021-09-04

**Authors:** Amorn Vijitpavan, Yanin Kooncharoensuk

**Affiliations:** Department of Anesthesiology, Faculty of Medicine Ramathibodi Hospital, Mahidol University, 270 Rama VI Rd, Ratchathewi, Bangkok, 10400 Thailand

**Keywords:** High flow, Nasal cannula, Intravenous sedation, Oxygenation, Ventilation, Desaturation

## Abstract

**Background:**

The dead space washout and provision of some level of positive airway pressure by nasal high-flow (NHF) cannula could improve the efficiency of ventilation, oxygenation and maintenance of the upper airway in patients undergoing deep sedation. This study aimed to compare the incidences of events represented oxygenation and ventilation, i.e. desaturation and upper airway obstruction, and arterial blood gas (ABG) levels between using NHF cannula and conventional nasal cannula (NC2) during deep sedation of adult surgical patients.

**Methods:**

In this prospective randomized single-blinded study, the patients who were 20–80 years old, ASA physical status of 1 to 3, scheduled for surgery under intravenous sedation (IVS) were included. The patients were randomly divided into two groups, i.e., NC2 or NHF groups. Propofol infusion was given to maintain deep sedation. The desaturation (SpO_2_ < 92%) and upper airway obstruction events (presence of snoring with paradoxical breathing) after application of NC2 or NHF were observed and collected. Other outcomes included interventions during IVS, such as jaw lifting or airway instrument insertion, mouth dryness, and post- ABG analyses were also collected and compared.

**Results:**

Thirty-six patients undergoing endovascular surgery were randomized into NC2 or NHF groups (18 in each group). No patients dropped out of the study. There were no significant differences in age, sex, ASA physical status, pre-sedation and pre-application of NC2 and NHF ABG analyses (PaO_2_, SaO_2_, pH, PaCO_2_, and PaO_2_/FiO_2_). NHF group had significantly lower in the incidence of desaturation [5 (27.78%) vs 13 (72.22%), *p* = 0.008], upper airway obstruction [4 (22.22%) vs 13 (72.22%), *p* = 0.003], and airway maneuver [6 (33.33%) vs 13 (72.22%), *p* = 0.019] than NC2 group. There were no differences in the ABG analyses after oxygen supplementation and no significant differences in mouth dryness between groups.

**Conclusion:**

The use of NHF cannula in patients undergoing endovascular surgery under deep sedation reduced desaturation events and required fewer airway interventions than NC2 with no difference in arterial blood gas analyses and mouth dryness.

## Introduction

Intravenous sedation is widely performed to sedate patients for many pain-free procedures. According to the American Society of Anesthesiologists (ASA), three levels of sedation are defined by responsiveness of the patient and abilities of the patient to maintain the airways and ventilation. With deep sedation, the upper airway muscles relax and cause upper airway obstruction, which may require some degree of an airway or ventilatory support [1]. Therefore, several techniques, such as chin lift, anterior displacement of the mandible, and airway device insertion, can be applied to improve airway patency [2]. Additionally, oxygen supplementation via multiple equipment and positive pressure ventilation via a face mask are performed. Adverse effects from these maneuvers may interrupt the depth of sedation and cause gastric inflation from positive pressure ventilation via a face mask that can lead to nausea or vomiting post-operation. Recently, a nasal high-f low (NHF) cannula was reported to have beneficial effects in patients with obstructive sleep apnea [3], chronic obstructive pulmonary disease [4]. Several studies also demonstrated that the use of an NHF cannula could reduce hypoxia or desaturation events with good tolerance or minimal adverse events in the patients undergoing endoscopic retrograde cholangiography (ERCP) [5], bronchoscopy [6] and gastroscopy [7], and dental treatment [8] under sedation. A previous randomized controlled trial (RCT) showed benefit and safety of the NHF oxygen therapy in hypoxemic patients after receiving cardiothoracic surgery [9] and a prospective cohort studies shown that NHF cannula was a useful tool as an adjuvant or main oxygen therapy during induction of general anesthesia, maintenance of deep intraoperative sedation, and during early postoperative care [10]. 

An NHF cannula can deliver positive airway pressure (PAP) in the upper airway without the use of an uncomfortable face or nasal mask compared with a conventional PAP machine. Mean nasopharyngeal pressure during NHF therapy increases as flow increases [11] and PAP created by an NHF depends on whether the person is breathing with the mouth open or closed and the flow rate [12]. Hypopharyngeal pressure increases with an increase in gas flow rate. The mean airway pressure is as high as 7.1 cmH_2_O when oxygen flow increases to 50 L/minute [13]. Moreover, patients can comfortably breathe warmed and fully humidified air, even when the flow rate is increased up to 50 L/minute. The gas could be humidified with a heated water chamber immediately before delivery to the patient [13]. Dead space washout and provision of some level of positive airway pressure by NHF device can improve efficiency of ventilation, oxygenation and self-maintaining of the upper airway [14]. However, there was no randomized controlled studies to demonstrate that NHF could improve oxygenation and ventilation in endovascular surgery patients undergoing intravenous deep sedation when compared with NC2.

Therefore, we conducted a prospective, randomized, controlled trial in inpatients who planned to have surgery under IVS, to demonstrate whether using an NHF cannula was able to maintain oxygenation and ventilation during deep sedation compared with a conventional nasal cannula (NC2) at the same fraction of inspired oxygen (FiO_2_).

## Methods

### Study design and participants

This prospective single-blinded randomized controlled study was approved by the Committee on Human Rights Related to Research Involving Human Subjects, Faculty of Medicine Ramathibodi Hospital, Mahidol University (Approval Certificate ID: MURA 2017/90. Protocol ID 02–60-12, approval on 30/03/2017). The study was registered on Thai Clinal Trial Registry http://www.clinicaltrials.in.th (Study ID: TCTR20201007002, Registration date 01/11/2018). This study was retrospectively registered in the registry because it was not a requirement for the approval of the protocol by the Committee on Human Rights Related to Research Involving Human Subjects of the Faculty of Medicine. The study was carried out in accordance with the Declaration of Helsinki and the Conference on Harmonization Guidelines for Good Clinical Practice and adhered to Consolidated Standards of Reporting Trials (CONSORT) guidelines [15].

Written informed consent was obtained from all participants prior to participation in this study. We recruited 20–80-year-old patients with an American Society of Anesthesiologists (ASA) physical status classification of 1 to 3 who were scheduled for surgery under IVS. The patients with known allergies to the anesthetic drugs used in the study and those with known unstable hemodynamics were excluded from the study.

Eligible patients were randomly divided into 2 groups. The patients in Group 1 received conventional nasal cannula with oxygen flow at 2 L/minute (Fraction of inspired oxygen (FiO_2_): 0.28; NC2 group) while the patients in Group 2 were administered nasal high flow (NHF) (Optiflow; Fisher & Paykel Healthcare, Auckland, New Zealand) therapy at a flow rate of 60 L/minute (FiO_2_: 0.28; NHF group). All gases for patients using an NHF cannula were administered through a humidifier and breathing circuits (AIRVO™ 2 System; Fisher & Paykel Healthcare). The randomization was performed with sealed envelope randomization services (available at http://www.sealedenvelope.com) with allocation ratio of 1:1 and block size of 2.

### Anesthesia and measurements

The primary outcome was the occurrence of desaturation defined as an SpO_2_ ≤ 92% (measured by pulse oximetry) after application of NC2 or NHF. The secondary outcomes were the occurrence of upper airway obstruction event defined as the present of snoring with paradoxical breathing, interventions during IVS, such as jaw lifting or airway instrument insertion, and arterial blood gas analyses after application of NC2 or NHF, and mouth dryness.

For anesthesia, an intravenous catheter was inserted with any sized catheter and Acetate Ringer’s solution was started at 60 mL/kg. Routine monitoring included noninvasive blood pressure, pulse rate, and pulse oximetry (SpO_2_), and electrocardiography (lead II) was performed before induction of anesthesia. Before starting sedation, an arterial line was inserted into a radial artery under local anesthesia (with 1% lidocaine without adrenaline) and then the first arterial blood sample was collected. The patient’s head was then repositioned by using rolled drapes to support the posterior neck and shoulder. Subsequently, hypnosis was induced by the administration of propofol using a target-controlled infusion system. The target blood concentration was maintained at 2 µg/mL to obtain an adequate deep level of sedation [16, 17]. The second arterial blood sample was then collected 15 min after propofol infusion to ensure the adequate depth of sedation. Administration of oxygen was initiated via NC2 or NCF cannula depending on the group assigned after an adequate depth of sedation was achieved. The definition of deep sedation was that the patients cannot be easily aroused but still had respond purposefully following repeated or painful stimulation [1]. In this study, we assessed the deep sedation by using verbal commands and shaking shoulders. The deeply sedated patients must not have any response to verbal commands but still have a response to shaking shoulders. The deep sedation was periodically assessed throughout the surgical procedure. The third arterial blood sample was collected after applying oxygen for 15 min. The response to surgical painful stimuli was got rid by infiltrating with 10 ml of 1% xylocaine at surgical site.

Anesthesiologists carefully monitored for upper airway obstruction by listening to breathing sounds and observing chest wall movement. Systolic and diastolic blood pressure, heart rate, and arterial hemoglobin saturation (SpO_2_) were measured and recorded every 5 min throughout the study. When systolic blood pressure decreased by 20% from baseline or was less than 100 mmHg, 3 to 6 mg of ephedrine was administrated. When SpO_2_ decreased below 92%, an intervention, such as jaw lifting or anterior mandibular displacement, was performed by the anesthesiologist and recorded. If SpO_2_ was still less than 90% in any period of interventions, oxygen was applied by a nasal cannula/NHF cannula or FiO_2_ was temporarily increased in case of both devices already be used. An oropharyngeal/nasopharyngeal airway was inserted when the upper airways could not be maintained by both devices. A nasopharyngeal airway was used first, and if upper airway obstruction still occurred, an oropharyngeal airway was applied. Positive pressure ventilation (PPV) via a face mask was performed when SpO_2_ was less than 88%. Taking blood samples was delayed for 15 min after positive ventilation and/or increased FiO_2_ was intervened. The target blood concentration was not changed until the surgical procedure was completed. Finally, the patients’ mouth dryness was evaluated by an interview while the patients did not know which study group they were in.

### Statistical analysis

The approximate rate of desaturation under intravenous sedation in a previous study was 40% [5, 18] in the patients receiving oxygen through conventional nasal cannula. The desaturation rates in the patients receiving oxygen through the nasal high-flow system during sedation in various condition from previous studies were ranged 0 to approximately 20% [5, 6, 19, 20]. We expected that the incidence of desaturation under the intravenous deep sedation in our study would be 40% in NC2 group and 2% in NHF group. Power and Sample Size Calculation Program (PS) version 3.1.2 was used to estimate the sample size with the ratio of NC5 to NHF subjects of 1:1. At least 17 subjects in each group were needed to be able to reject the null hypothesis that the desaturation rate during IVS between NC2 and NHF groups are equal with a power of 80% and a type I error probability of 0.05. Considering a dropout rate of 5% due to short term follow-up, the sample size required was 18 patients per group.

All analyses were performed on an intention-to-treat basis using IBM SPSS Statistics for Windows, Version 20.0. (IBM Corp, Armonk, NY, USA). Baseline categorical characteristics are described as number with percentage and/or 95% confidence interval (95%CI) and the quantitative variables as mean ± standard deviation (SD). Dichotomous variables were compared with the χ^2^ test or Fisher’s test, as appropriate. Continuous variables were compared with the t-test or Wilcoxon rank-sum test as appropriate. A *P*-value of less than 0.05 was considered statistically significant for all tests.

## Results

### Baseline demographic and clinical characteristics

**Fig. 1:**
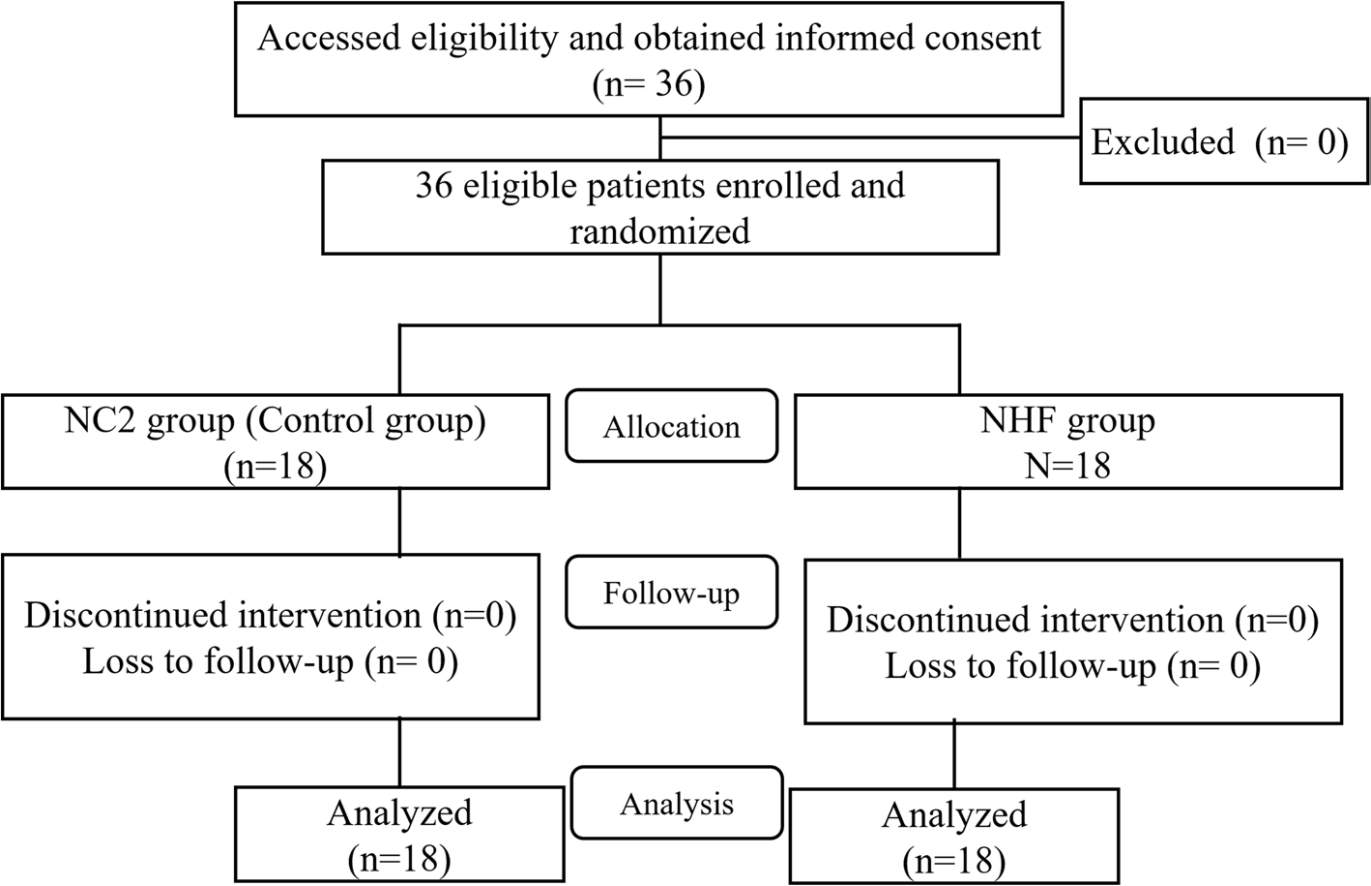
Participant Flow

From April 2017 to February 2018, there were 36 eligible patients scheduled for surgical treatment under IVS. All patients screened and enrolled were patients undergoing endovascular surgery. The patients were randomized to either NC2 or NHF group using an equal allocation. No patients dropped out of the study (Fig. 1 Participant Flow). Thus, all randomized patients were included in the analyses. There were no significant differences in age, sex, male/female, body mass index, ASA physical status, having obstructive sleep apnea, and pre-sedation blood gases between the two groups (Table 1).

**Table 1:** Baseline characteristics

	**NC2 group (*n* = 18)**	**NHF group (*n* = 18)**	***P* value**
**Age (years)**	67.33 ± 8.83	67.44 ± 12.84	0.976
**Sex, male/female, n**	11/7	11/7	1.000
**Body mass index (kg/m**^**2**^**)**	22.85 ± 3.38	24.29 ± 4.56	0.291
**ASA physical status**			1.000
I	1 (5.56%)	1 (5.56%)	
II	1 (5.56%)	1 (5.56%)	
III	16 (88.89%)	16 (88.89%)	
**Obstructive sleep apnea**	1 (5.56%)	0 (0%)	1.000
**Pre-sedation blood gases**			
pH	7.45 ± 0.03	7.42 ± 0.07	0.121
PaCO_2_	38.15 ± 3.25	40.34 ± 4.13	0.086
PaO_2_	78.81 ± 16.88	79.37 ± 12.87	0.912
SaO_2_	95.78 ± 1.93	96.49 ± 2.61	0.359
PaO_2_/FiO_2_	368.85 ± 84.5	372.08 ± 63.41	0.898
**Endovascular surgery, n (%)**	18 (100%)	18 (100%)	NA

Data are mean ± standard deviation or n (%)

*Abbreviations*: *NA* Not applicable, *NHF* Nasal high flow, *NC2* Conventional nasal cannula, *PaCO*_*2*_ Partial pressure of carbon dioxide in arterial blood, *PaO*_*2*_ Partial pressure of oxygen in arterial blood, *SaO*_*2*_ Oxygen saturation in arterial blood, *FiO*_*2*_ Fraction of inspired oxygen, *FiO*_*2*_ Fraction of inspired oxygen

### Outcomes

NC2 group had a significantly higher incidence of desaturation than the NHF group [13 (72.22%) vs. 5 (27.78%); *p* = 0.008]. Besides, there was a significantly higher incidence of upper airway obstruction in the NC2 group than in the NHF group [13 (72.22%) vs. 4 (22.22); *p* = 0.003]. The NC2 group required interventions more frequently when compared with the NHF group. However, there was no statistically significant difference in instrument insertion between groups. One of 18 patients in the NHF group required PPV via a face mask during IVS. There was no significant difference in the rate of mucosal dryness between the two groups (Table 2).

**Table 2:** Complications, airway intervention and instrument insertion

	**NC2 group (*n* = 18)**	**NHF group (*n* = 18)**	***P* value**
**Complications**			
Desaturation (SpO_2_ < 92%) during IVS^a^	13 (72.22%) [46.52, 90.31%]	5 (27.78%) [9.69, 53.48]	0.008*
Upper airway obstruction during IVS^b^	13 (72.22%) [46.52, 90.31%]	4 (22.22%) [6.41, 47.63%]	0.003*
Mucosal dryness	2 (11.11%) [1.38, 34.71%]	1 (5.56%) [0.14, 27.29%]	1.000
**Required interventions**			
Airway maneuver^c^	13 (72.22%) [46.52, 90.31%]	6 (33.33%) [13.34, 59.01%]	0.019*
PPV requirement^c^	0 (0%)	1 (5.56%) [0.14, 27.29%]	1.000
**Instrument insertion**			
Nasopharyngeal airway	6 (33.33%) [13.34, 59.01%]	3 (16.67%) [3.58, 41.41%]	0.443
Oropharyngeal airway	2 (11.11%) [1.38, 34.71%]	1 (5.56%) [0.14, 27.29%]	1.000

Parentheses are percentages and values in square brackets represent 95% confidence interval (CI)

*Abbreviations*: *IVS* Intravenous sedation (IVS), *PPV* Positive pressure ventilation

^*^Significant at *P* value < 0.05

^a^Desaturation events after application of NC or NHF during deep sedation

^b^Upper airway obstruction after application of NC or NHF during deep sedation. The Upper airway obstruction during IVS defined as the present of snoring with paradoxical breathing

^c^One patient in NHF group received airway maneuver with PPV without desaturation and upper airway obstruction during IVS because the patient had apnea

The arterial partial pressure of oxygen (PaO_2_) and the PaO_2_/FiO_2_ (PF ratio) after oxygen supplementation were not significantly different between the two groups. The partial pressure of carbon dioxide (PaCO_2_) was also not significantly different between the groups (Table 3).

**Table 3:** Arterial blood gas analysis between groups (*n* = 18 in each group)

	**Baseline**		**Post-sedation**^**a**^			**Post-sedation with oxygen device intervention**^**b**^		
	NC2 group	NHF group	NC2 group	NHF group	*P* value	NC2 group	NHF group	*P* value
**PaO**_**2**_	78.81 ± 16.88	79.37 ± 12.87	63.14 ± 14.75	62.56 ± 25.12	0.934	110.16 ± 52.77	109.32 ± 28.67	0.954
**SaO**_**2**_	95.78 ± 1.93	96.49 ± 2.61	90.54 ± 5.88	87.45 ± 12.24	0.344	95.97 ± 3.72	97.34 ± 2.51	0.205
**pH**	7.45 ± 0.03	7.42 ± 0.07	7.41 ± 0.06	7.4 ± 0.06	0.394	7.38 ± 0.04	7.38 ± 0.05	0.705
**PaCO**_**2**_	38.15 ± 3.25	40.34 ± 4.13	42.48 ± 3.67	44.84 ± 4.79	0.107	45.35 ± 5.69	46.18 ± 5.56	0.660
**PaO**_**2**_**/FiO**_**2**_	368.85 ± 84.5	372.08 ± 63.41	296.15 ± 71.07	293.21 ± 119.85	0.929	379.34 ± 183.11	385.71 ± 105.43	0.899

Data are mean ± standard deviation (SD)

*Abbreviations*: *NHF* Nasal high flow, *NC2* Conventional nasal cannula, *PaCO*_*2*_ Partial pressure of carbon dioxide in arterial blood, *PaO*_*2*_ Partial pressure of oxygen in arterial blood, *SaO*_*2*_ Oxygen saturation in arterial blood, *FiO*_*2*_ Fraction of inspired oxygen, *FiO*_*2*_ Fraction of inspired oxygen

^a^Blood collection 15 min after propofol infusion and before application of nasal conventional or NHF cannula

^b^After application of NC2 or NHF

## Discussion

In this study, we used propofol infusion to maintain deep sedation during endovascular surgery. Relaxation of the tongue and pharyngeal muscles causes narrowing or closing of the upper airway space, resulting in upper airway obstruction in patients under deep sedation. Although a simple airway maneuver, such as head tilt, chin lift, or jaw thrust, may be effective to relieve this obstruction, it is often inconvenient to perform during the surgical period. In our study, the NHF system helped reduce the need to open airway obstruction and reduce the incidence of desaturation compared with a conventional nasal cannula. Improving gas exchange could be achieved by an NHF cannula-induced reduction in rebreathed CO_2_ volume by clearance of dead space [21, 22] and an increase in end-expiratory lung volume [23].

NHF can also generate PAP. Many studies on NHF cannulas have shown that this device could generate mean pressure in the pharyngeal space up to 7.1 cmH_2_O when flow was delivered up to 50 L/minute [11–13, 24]. In this study, the NHF group were administered at a flow rate of 60 L/minute that higher than the previous studies [11–13, 24]. The higher rate might be helpful in relieving upper airway obstruction and reduce the requirement for performing airway maneuvers in deeply sedated patients in NHF group. The Starling resistor model of the upper airway is used to explain the collapsible (pharyngeal) segment between two rigid tubes (nasal and trachea) [25]. If the airway pressure in upstream is more than critical closing pressure (pressure required to collapse the airway), the obstruction will be relieved and airflow can pass through the lower airways. The study by Lin Y et al. [7] in outpatients undergoing routine gastroscopy with propofol sedation that used the nasal high flow (NHF) therapy at the flow rate up to 60 L/minute showed the incidence of hypoxia of 0% in NHF group while the desaturation rate in NHF group in the present study was 27.78%. The higher desaturation rate might be from the differences in oxygen concentration (FiO_2_) and patient positioning. FiO_2_ in our study was 0.28 and all patients were in supine position, whereas the study by Lin et al. applied FiO_2_ of 1.0 and all patients were in lateral position. In addition, the study patient characteristics were also different. Almost all patients in our study were ASA physical status grade 3 while the study by Lin Y et al. included the patients with ASA physical status grade 1 to 2 only.

A previous study [26] showed that an upstream pressure equal to 11.8 ± 2.7 cmH_2_O was sufficient to maintain airway patency. Mathru M et al. found that nasal continuous PAP (CPAP) can restore the patency of the pharyngeal airway in patients sedated with propofol [27]. In our study, there were 6/18 patients in the NHF group required an airway maneuver. A reason of airway maneuver requirements could be from insufficient PAP level generated by NHF to open the collapsed airway, which might require upstream pressure of more than 12 cmH_2_O, as mentioned above. The PAP created by NHF depends on whether the person is breathing with the mouth opened or closed and the flow rate used.

In this study, there was no control of the mouth position. Additionally, because sedated patients might have had some mechanical obstruction at the nasopharynx, base of the tongue, and hypopharynx, the NHF could not combat the resistance from those obstructions. Therefore, the airflow proximally leaked from the airway obstruction area. Subsequently, the NHF system was unable to maintain PAP all of the time. In the present study, although the incidence of desaturation and upper airway obstruction in NHF group was significantly lower than NC2 group, however, some patients in NHF group required combination of the interventions. Of the 5 patients having desaturation in NHF group, 3 patients had airflow leak and required both the airway maneuver and airway instrument insertion to maintain SpO_2_ over 92%. Two of the 3 patients required nasopharyngeal airway insertion only and one patient needed both nasopharyngeal and oropharyngeal airway insertion. In addition, one patient in NHF group experienced apnea during the deep sedation. Recent studies demonstrated that transnasal humidified rapid insufflation ventilatory exchange (THRIVE), an oxygenation technique that delivers continuous, warm and humidified oxygen at a high flow rate (up to 120 L min^−1^) [28] via high flow nasal cannula could prolong the safe apnea time both adults [29] and children [30]. The THRIVE technique is easily implemented method to achieve oxygenation and ventilation without an invasive device [31]. Further research into THRIVE by comparing with current oxygenation technique with NHF cannula is required to demonstrate the benefit of THRIVE in the patients undergoing surgery under deep sedation.

In this study, post-intervention arterial blood gas analysis showed no significant difference in partial pressure of oxygen (PaO_2_) and carbondioxide (PaCO_2_) between using the NHF system and a conventional nasal cannula. Although PaO_2_ and PaCO_2_ were not correlated with clinical desaturation and the need to perform airway interventions, which had a significantly higher incidence in the NC2 group than in the NHF group. The reason for this finding could be because there were patients with severe hypoxemia in the NC2 group. Therefore, we had to prevent further desaturation by performing an airway maneuver and inserting an airway device before collecting blood samples. For this reason, an accurate measurement of oxygenation and ventilation could not be obtained at this time.

In the NC2 group, two patients had an extremely high PaO_2_ and PF ratio, which caused an increase in overall oxygenation. As a result, oxygenation in the NC2 group was not significantly different compared with that in the NHF group. Generally, a conventional nasal cannula should only be used with an oxygen flow rate of 6L/minute because exceeding 6 L/minute causes dryness of the nasal mucosa. A previous study reported that an oxygen flow rate of up to 50 L/minute could be delivered by a conventional nasal cannula, but only when the gas was optimally warmed and humidified [32]. In our study, only one patient complained about airway dryness, and it did not lead to low satisfaction by the patient while he was applied oxygen via an NHF cannula.

A previous randomized controlled study showed benefit and safety of the NHF oxygen therapy in hypoxemic patients after receiving cardiothoracic surgery [9] and a prospective cohort studies shown that NHF cannula was a useful tool as an adjuvant or main oxygen therapy during induction of general anesthesia, maintenance of deep intraoperative sedation, and during early postoperative care [10]. However, our study would be the first randomized controlled study comparing nasal high flow cannula versus conventional nasal cannula in the patients undergoing endovascular under IVS. Although the sample size was small, the significant differences in desaturation, upper airway obstruction, and airway maneuver were detected. The study with larger sample size could give more precise estimates of effects (narrower confidence intervals). Despite the positive results, this study has some limitations. We could not properly control the timing of drawing arterial blood gas because of ethical issues about the safety of patients after desaturation occurred. The apnea time and the length of desaturation was not collected and the screening tools for the risk of obstructive sleep apnea (OSA) e.g., STOP-Bang Questionnaire was not applied. Moreover, further studies should measure the nasopharyngeal pressure to clarify if the NHF system was able to maintain PAP or not.

In conclusion, the use of NHF cannula in patients undergoing endovascular surgery under deep sedation reduced desaturation events and required fewer airway interventions than NC2 with no difference in arterial blood gas analyses and mouth dryness.

## Data Availability

The unidentifiable datasets used and/or analyzed during the current study are available from the corresponding author on reasonable request.
